# Warthy-Basaloid Squamous Cell Carcinoma of Penile – Case Report

**DOI:** 10.3389/fonc.2021.765640

**Published:** 2021-11-11

**Authors:** Natalia Domian, Grzegorz Młynarczyk, Irena Kasacka

**Affiliations:** ^1^ Department of Histology and Cytophysiology, Medical University of Białystok, Białystok, Poland; ^2^ Department of Urology, Medical University of Białystok, Białystok, Poland

**Keywords:** penile squamous cell carcinoma, warty carcinoma, basaloid carcinoma, human papillomavirus, adhesion molecules

## Abstract

**Objective:**

The aim of the study was to present a case of penile squamous cell carcinoma and immunohistochemical identification and evaluation of E-cadherin and β-catenin expression.

**Methods:**

We are presenting a 70-year old man with a variant of penile squamous cell carcinoma with mixed warty and basaloid features. After diagnosis, the patient underwent partial penectomy. Samples taken from the material after surgery were subjected to basic histological staining and immunohistochemical identification of E-cadherin and β-catenin. A Real-time PCR study was conducted to investigate the expression of E-cadherin and β-catenin.

**Results:**

Routine histopathological examinations revealed the characteristic features of warty-basaloid squamous cell carcinoma. In the case studied, a positive immunohistochemical reaction was observed for E-cadherin and β-catenin. QRT-PCR analysis showed a statistically significant decrease in E-cadherin expression in tumor samples compared to healthy tissue. In contrast, expression of the gene encoding β-catenin was slightly higher in tumor samples compared to normal tissue.

**Conclusions:**

The reduced level of the complex of adhesive elements, E-cadherin-β-catenin, disturbs cell differentiation, promotes a more invasive phenotype-stromal infiltration and the formation of distant metastases. In the described case of the penile tumor, a decrease in E-cadherin expression was noted, which could be related to the occurrence of neoplastic infiltration of the spongy body space. In summary, E-cadherin and β-catenin expression and the immunoreactivity of these proteins are expressed at different levels in tumor cells and in penile interstitial cells. Regulation of expression during various physiological and pathophysiological processes indicates a potentially important role of E-cadherin and β-catenin in cell proliferation and adhesion.

## Introduction

The human papillomavirus (HPV) is the most common viral infection of the reproductive tract. Wherein, it does not cause systemic infection, but a local one and most of them are asymptomatic and resolves spontaneously. Persistent infections cause changes to the skin (warts) and can lead to the development of cancer. HPV infects epithelial cells of the skin and mucous membranes, and their development cycle is related to the differentiation of infected cells. HPV infection is closely related to penile cancer, but the relationship between HPV infection and cancer formation is not fully understood (1, 2).

Penile cancer is a fairly rare cancer with approximately 26 000 cases diagnosed worldwide each year. Most penis cancers are squamous cell carcinomas, but there is a wide spectrum of histological subtypes. Numerous observations indicate the participation of HPV in the incidence of penile cancer. The role of HPV has been confirmed in the etiology of squamous cell carcinoma of the penis in men, but it has been established that other factors are also involved in this process (3, 4).

There are three types of penile squamous cell carcinoma, usually associated with the HPV: warty-basaloid, warty carcinomas and basaloid. In invasive penile tumors, papillary-basal carcinomas were most often associated with HPV. It is supposed that these viruses are present only in the initial stage of the precancerous state, and later, along with the development of neoplastic changes, their genes cannot be found. So they are undetectable at this stage (5).

HPV infection is not able to induce complete neoplastic transformation. This process requires the participation of other factors, such as the escape of cells from the control of the immune system, changes in the expression of viral genes, and subsequent genetic changes. Studies have shown that an interaction can occur between the HPV oncoprotein and p53, which leads to the inactivation of the p53 gene, which is a negative regulator of tumor cell growth. In response to DNA damage, it may arrest the G1 cell cycle and/or apoptosis. Research is constantly being carried out to identify successive genetic changes related to the oncogenesis process stimulated by HPV, or involving HPV (6, 7).

In the course of tumor progression, epithelial cells, in the course of oncogenesis, begin to acquire the features of mesenchymal cells. It is associated with the loss or reduction of intercellular connections, and weakened interaction with the basement membrane. Intercellular junctions maintain apical-basal polarization, maintain tissue integrity, and enable interaction and signal transmission between cells and between the extracellular matrix. Weak cell adherence may lead to impaired control of the cell cycle, separation of single cells from the primary site, which creates conditions for the formation of neoplastic metastases (8).

One of the basic adhesion molecules responsible for the formation of intercellular connections and the mutual recognition of cells is E-cadherin. As a result of its loss, the accumulation of β-catenin in the cytoplasm of the cell occurs and its translocation to the cell nucleus, where it regulates the transcription of many genes involved in the proliferation and differentiation of cells.

E-cadherin expression reduction or function shutdown is associated with the loss of intercellular connections, proper polarity and the acquisition of the ability to migrate and invade, which are key phenomena responsible for the progression of neoplastic disease (8, 9).

Warthy-basal cell carcinoma of the penis is a rare disease that is clinically and pathologically diverse. New factors involved in the cancer progression are still being searched for. E-cadherin and B-catenin could prove to be important biomarkers that have not yet been assessed together in this type of cancer.

The aim of the study was to present the case of penile squamous cell carcinoma and immunohistochemical identification and evaluation of E-cadherin and B-catenin expression. This study is rare and contains new data.

## Material and Methods

### Clinical Presentation

We are presenting a 70-year old male patient who noticed a change in the area of the glans penis in 2017. In July 2019, a biopsy was performed. The histopathological analysis of this biopsy was found (hist-pat. *Planocellulare invasivum*). The patient was proposed a partial penile amputation, for which the patient did not consent.

He was hospitalized only at the beginning of 2021 at the Clinical Hospital in Białystok, the lesion has increased by about 10-15 mm. Before the surgery color Doppler ultrasound of the penis was performed. There was a suspicion of cavernous infiltration. This was confirmed in histopathomorphology. Patient underwent a partial penectomy procedure.

The study protocol was approved by the Bioethics Committee, Medical University of Bialystok (R-I-002/282/2019) and prior written informed consent was obtained from patient.

### Clinicopathologic Features

The distal part of the penis measures 5 x 3.2 x 3.5 cm. In the area of ​​the glans and foreskin, there is an exophytic ulcerative tumor 3.7x2x1.5 cm. On the glans cross-sections, a whitish infiltrate is present, covering the distal part of the corpora cavernosa and penetrating superficially into the left corpora cavernosa.

### Pathomorphological Diagnosis

Histological type: warthy-basaloid squamous cell carcinoma. Histological maturity grade: G2 - moderately differentiated. The tumor invades the spongy body, the infiltration is at least 8 mm thick. There was no invasion of the perineural spaces. Pathomorphological stage (pTNM): pT2 pNx pMx. Inguinal nodes were not palpable.

The diagnosed variant of squamous cell carcinoma is typically associated with an HPV infection. P16 test - diffuse positive.

### Immunohistochemistry

Material were embedded in paraffin in a routine manner. The paraffin blocks were cut into 4 µm sections and attached to positively charged glass slides and stained in hematoxylin and eosin for general histological evaluation. Immunostaining was performed by the following protocol: sections were deparaffined and hydrated in pure alcohols. For antigen retrieval, the sections were subjected to pretreatment in pressure chamber and heated for 1 min at 21 psi at 125°C, using Target Retrieval Solution Citrate pH=6.0 (S 2369 Agilent Technologies, Inc. 5301 Stevens Creek Blvd Santa Clara, CA 95051, USA). After cooling down to room temperature, the sections were incubated with Dako REAL Peroxidase-Blocking Solution (S 2023 Agilent Technologies, Inc.) for 10 minutes to block endogenous peroxidase activity. The sections with the primary antibodies: β-catenin, (ab32572 Abcam, UK) and E-cadherin (ab76055 Abcam, UK) were incubated 24 hours at +4°C in a humidified chamber. The antibodies were previously diluted in Antibody Diluent Background Reducing (S 3022 Agilent Technologies, Inc.) in relation 1: 2 000 for β-catenin and 1: 500 for E-cadherin. Procedure was followed by incubation (1 hour) with secondary antibody (EnVision FLEX, High pH (Link), HRP. Rabbit/Mouse. (K800021-2 Agilent Technologies, Inc.). The bound antibodies were visualized by 1-min incubation with DAB Flex chromogen. The sections were finally counterstained in hematoxylin QS (H-3404, Vector Laboratories; Burlingame, CA), mounted and evaluated under light microscope. Appropriate washing with Wash Buffer (S 3006 Agilent Technologies, Inc.) was performed between each step (3 times for 2 minutes). Sections were dehydrated with absolute alcohol followed by xylene, and coverslipped with Entellan (Merck). The specificity of the antibodies was confirmed using a negative control, which involved replacing the antibodies with the Antibody Diluent (no staining).

### Real-Time PCR

Tumor and normal tissue samples taken from the material after partial penectomy were placed in an RNA-later solution. Total RNA was isolated using NucleoSpin^®^ RNA Isolation Kit (Machery-Nagel). Quantification and quality control of total RNA was determined using a spectrophotometer - NanoDrop 2000 (ThermoScientific). An aliquot of 1 µg of total RNA was reverse transcribed into cDNA using iScript™ Advanced cDNA Synthesis Kit for RT-qPCR (BIO-RAD). Synthesis of cDNA was performed in a final volume of 20 μl using an Thermal Cycler (Model SureCycler 8800, Aligent Technologies). For reverse transcription, the mixtures were incubated at 46°C for 20 min, then heated to 95°C for 1 min and finally cooled quickly at 4°C. Quantitative real-time PCR reactions were performed using Stratagene Mx3005P (Aligent Technologies) with the SsoAdvanced™ Universal SYBER^®^ Green Supermix (BIO-RAD). Specific primers for E-cadherin, β-catenin and GAPDH (GAPDH) were designed by BIO-RAD Company. The housekeeping gene GAPDH (GAPDH) was used as a reference gene for quantification. To determine the amounts of levels of test genes expression, standard curves were constructed for each gene separately with serially diluted PCR products. PCR products were obtained by cDNA amplification using specific primers as follows: E-cadherin (qHsaCEP0049339, BIO-RAD), β-catenin (qHsaCID0010363, BIO-RAD), and GAPDH (qHsaCED0038674, BIO-RAD). QRT-PCR was carried out in a doublet in a final volume of 20 μl under the following conditions: 2 min polymerase activation at 95°C, 5 s denaturation at 95°C, 30 s annealing at 60°C for 35 cycles. PCR reactions were checked, including no-RT-controls, omitting of templates, and melting curve to ensure only one product was amplified. The relative quantification of gene expression was determined by comparing Ct values using the ΔΔCt method. All results were normalized to GAPDH.

### Statistical Analysis

All data were analyzed for statistical significance using the Statistica version 12.0 computer software package. The mean values were computed automatically; significant differences were determined by one-way ANOVA test; p < 0.05 was considered significant.

## Results

**Figure 1:**
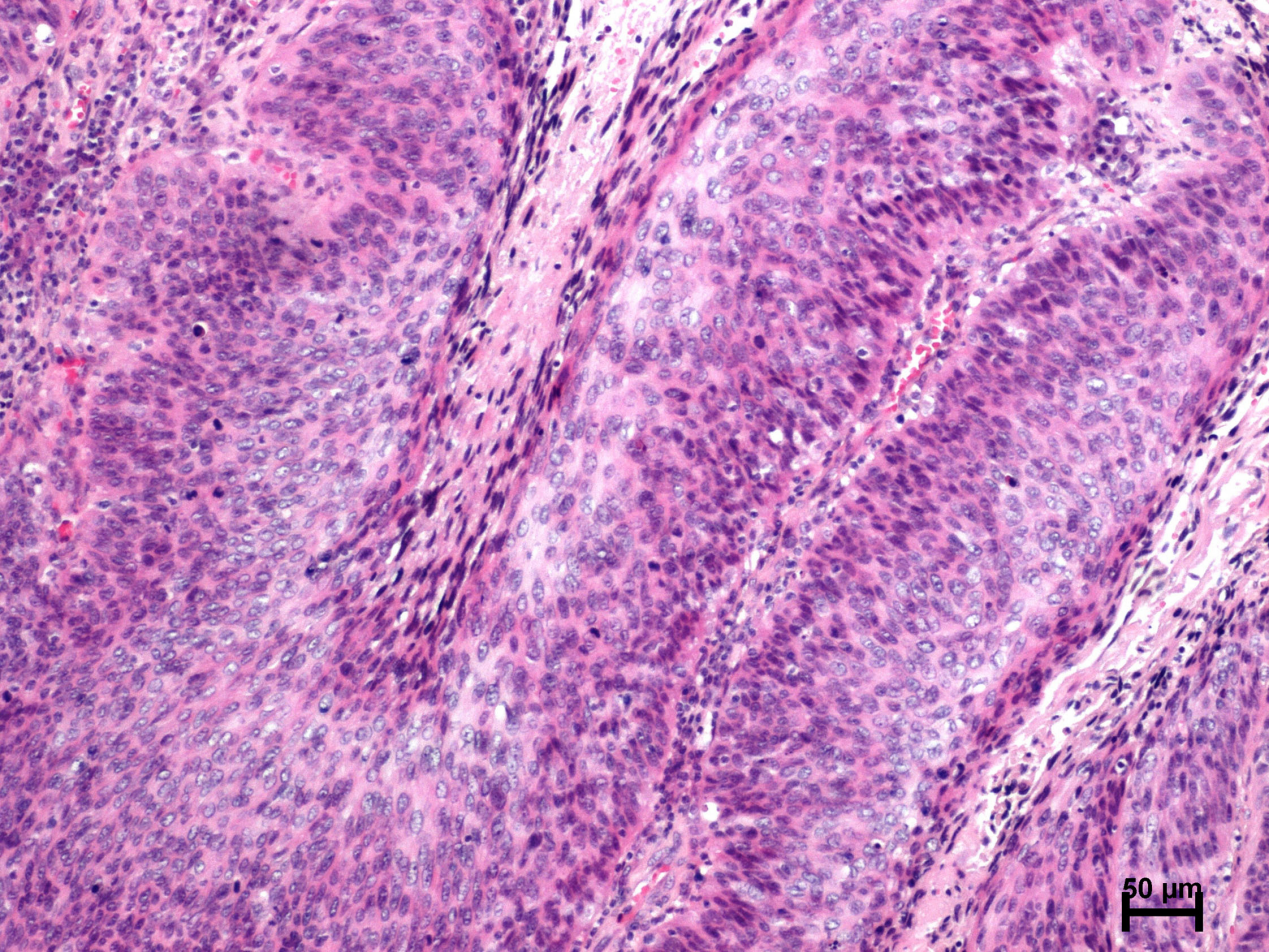
Microscopic features of warty–basaloid carcinoma (H+E).

Routine histopathological examinations showed the characteristic features of warty-basaloid squamous cell carcinoma (**Figure 1**). Invasive tumor nest with central atypical parakeratosis, pleomorphic koilocytosis and peripheral small and uniform cells with basaloid features. The papillae had conspicuous fibrovascular cores. Cells with basaloid features predominated, although rounded and spindle cells were also noted. An evident clear cell koilocytosis on surface was present in this case (**Figure 1**).

**Figure 2:**
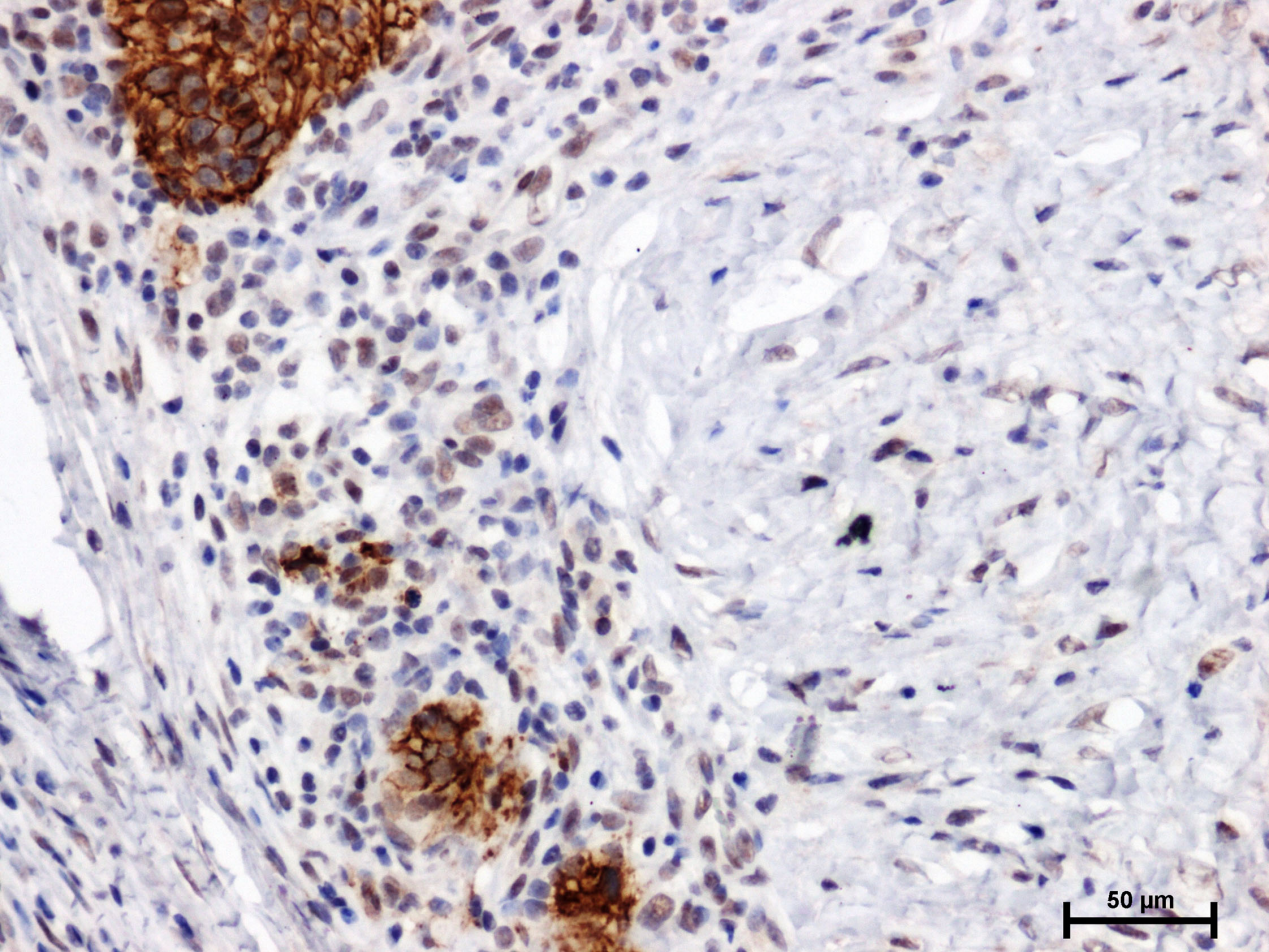
Immunoidentification of E-cadherin in warty–basaloid carcinoma of penile.

**Figure 3:**
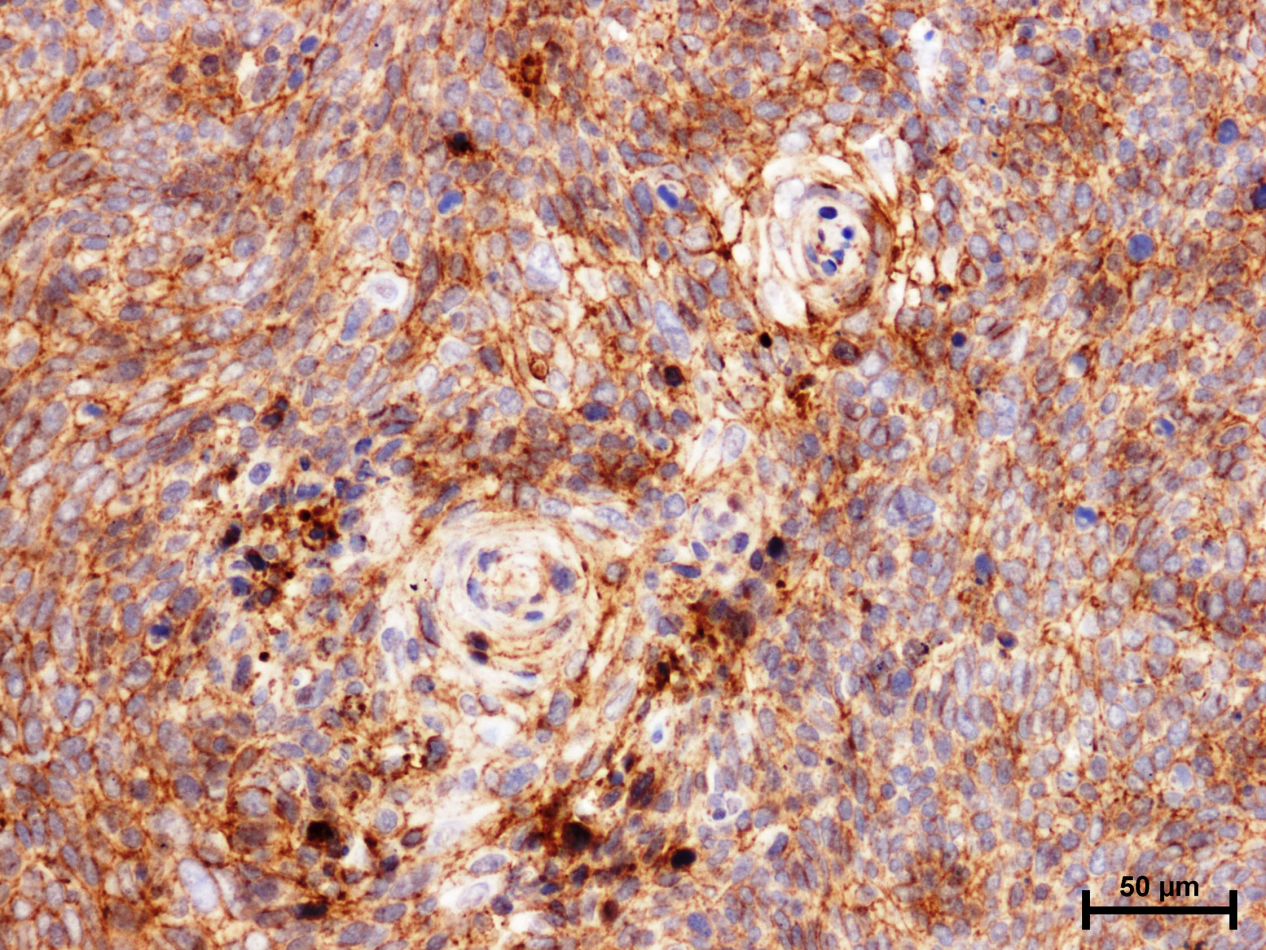
Immunoidentification of β-catenin in warty–basaloid carcinoma of penile.

A positive immunohistochemical reaction for E-cadherin and β-catenin was observed in the studied warty-basaloid squamous cell carcinoma case (**Figures 2**, **3**).

The immunoreactivity of E-cadherin in penile cancer neoplastic cells is mainly located in the cell membrane (**Figure 2**). Cells with a highly stained cell membrane were adjacent to weakly stained or negative cells (**Figure 2**).

In the attached photos (**Figure 3**) we observe the membrane immunoexpression of β-catenin, as well as the presence of β-catenin in the cytoplasmic compartment and translocation to the cell nucleus.

**Figure 4:**
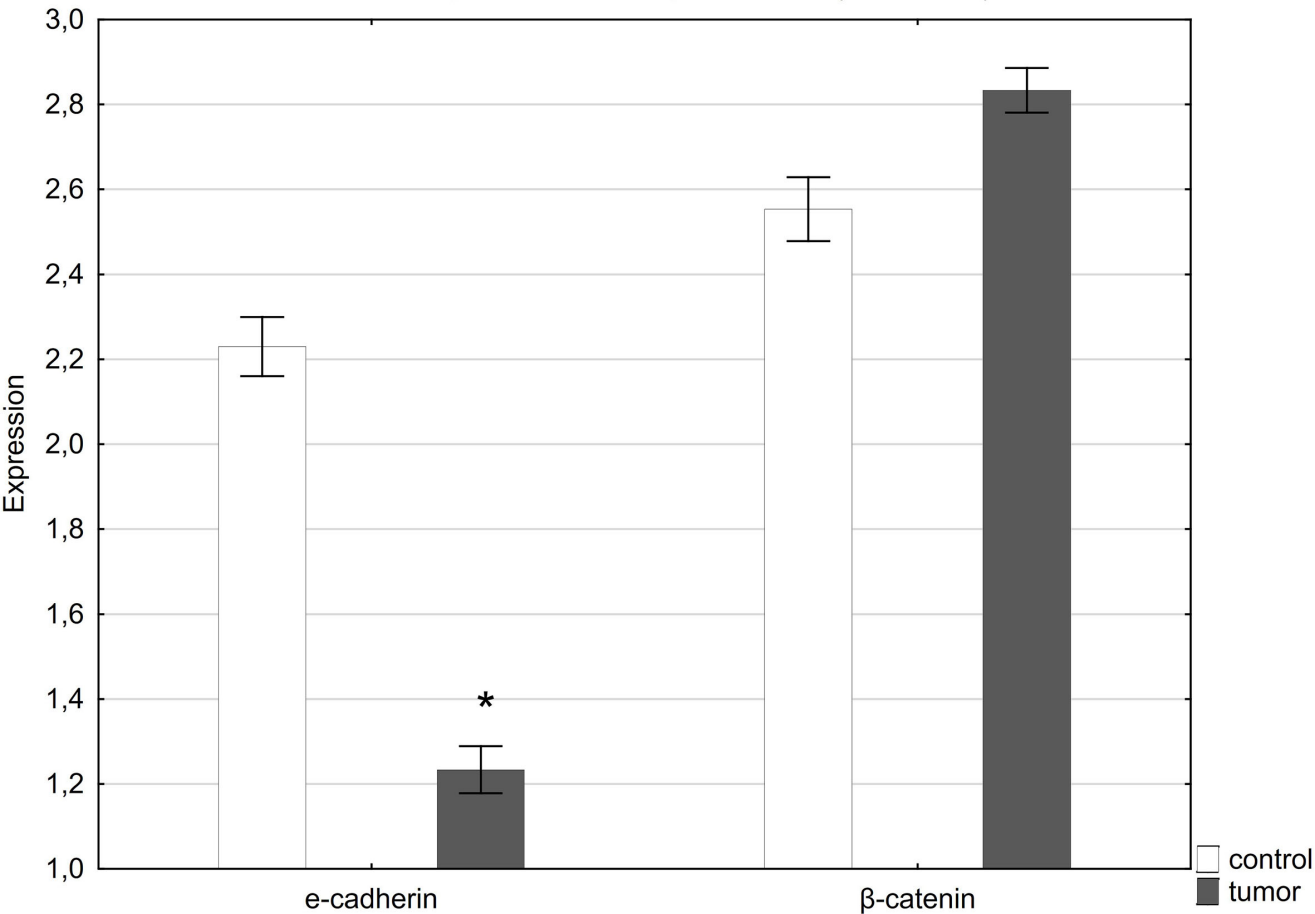
Expression of E-cadherin and β-catenin in normal penile tissue (control) and penile tumor. *p < 0.05 was considered significant.

QRT-PCR analysis showed a statistically significant decrease in E-cadherin expression in tumor samples compared to healthy tissue. In contrast, expression of the gene encoding β-catenin was slightly higher in tumor samples compared to normal tissue (**Figure 4**).

## Discussion

Penile cancer can follow various etiological pathways, one of them is associated with HPV infection, and the others are associated with chronic inflammation, phimosis, etc. The prevalence of different histological types of penile cancer varies, the most common type is papillary and/or basal cell carcinoma, less frequently keratinizing variants. The presence of cancer cells with basaloid features is strongly associated with the presence of HPV (5, 10, 11).

We describe a mixed neoplasm exhibiting warty and basaloid features. Considering the pathogenesis associated with the human papilloma virus, as well as mixed morphological forms, warty and basaloid carcinomas would represent the low- and high-grade ends of a clinicopathological spectrum (4, 12, 13).

E-cadherin, as a component of the E-cadherin-catenin adhesive complex, acts as an “invasion suppressor”. Reducing its level leads to a weakening of intercellular adhesive interactions, disrupts the integrity and structure of the tissue, and promotes the aggressive features of neoplastic cells - infiltration of the stroma, invasion of blood vessel and formation of metastasis (14, 15).

The decreased level of E-cadherin stimulates the proliferative activity of cells, as well as the process of epithelial-mesenchymal transformation, during which neoplastic cells lose the ability to form epithelial structures and develop a mesenchymal phenotype, which allows for cell dissociation, invasion of the environment and the formation of metastases (16).

In the described case of a penile tumor, a decrease in the expression of E-cadherin was noted, which could be related to the occurrence of a neoplastic infiltration of the spongy body space.

In normal epithelium, β-catenin is located in the cell membrane. Reduced membrane immunoexpression, as well as the presence of β-catenin in the cytoplasmic compartment and translocation to the cell nucleus are interpreted as indicators of decreased immunoexpression of this protein (17).

In the performed immunohistochemical reaction showing β-catenin in the examined penile tumor, positive membrane, cytoplasmic and nuclear reactions were observed. Furthermore, we found a slight increase in the expression of the gene encoding beta-catenin in the tumor tissue as compared to the control.

In over half of all cancer cases, such as breast cancer, leukemia, melanoma, colorectal cancer and liver cancer, β-catenin accumulates in the nucleus or the cytoplasm. Research has shown that β-catenin promotes the progression of tumors *via* suppressing the T-cell responses (18, 19).

Reduced level of E-cadherin-β-catenin adhesive complex elements disturbs cell differentiation, promotes a more invasive phenotype-stromal infiltration and the formation of distant metastases (8).

In conclusion, E-cadherin and β-catenin expression and the immunoreactivity of these proteins are expressed at different levels in tumor cells and in penile interstitial cells. Regulation of expression during various physiological and pathophysiological processes indicates a potentially important role of E-cadherin and β-catenin in cell proliferation and adhesion. The reduced level of E-cadherin-β-catenin, disturbs cell differentiation, promotes a more invasive phenotype-stromal infiltration and the formation of distant metastases.

## Data Availability Statement

The original contributions presented in the study are included in the article/supplementary material. Further inquiries can be directed to the corresponding author.

## Ethics Statement

The studies involving human participants were reviewed and approved by The Bioethics Committee, Medical University of Bialystok (R-I-002/282/2019). The patients/participants provided their written informed consent to participate in this study.

## Author Contributions

IK and ND conceived of and designed the experiments. ND and GM analyzed the data. IK, ND, and GM contributed reagents/materials/analysis tools. Writing – original draft preparation: ND. Writing – review and editing: IK. All authors contributed to the article and approved the submitted version.

## Funding

This work was supported by statutory funds from the Medical University of Bialystok SUB/2/DN/21/001/2232.

## Conflict of Interest

The authors declare that the research was conducted in the absence of any commercial or financial relationships that could be construed as a potential conflict of interest.

## Publisher’s Note

All claims expressed in this article are solely those of the authors and do not necessarily represent those of their affiliated organizations, or those of the publisher, the editors and the reviewers. Any product that may be evaluated in this article, or claim that may be made by its manufacturer, is not guaranteed or endorsed by the publisher.
